# Advances in the genetic characterization of the malaria vector, *Anopheles funestus,* and implications for improved surveillance and control

**DOI:** 10.1186/s12936-023-04662-8

**Published:** 2023-08-08

**Authors:** Joel O. Odero, Ismail H. Nambunga, Dimitri W. Wangrawa, Athanase Badolo, David Weetman, Lizette L. Koekemoer, Heather M. Ferguson, Fredros O. Okumu, Francesco Baldini

**Affiliations:** 1 Environmental Health and Ecological Sciences Department, Ifakara Health Institute, Ifakara, Tanzania; 2 Laboratoire d’Entomologie Fondamentale et Appliquée, Université Joseph ZEBRO, Ouagadougou, Burkina Faso; 3 School of Biodiversity, One Health, and Veterinary Medicine, University of Glasgow, Glasgow, G12 8QQ UK; 4 Department of Vector Biology, Liverpool School of Tropical Medicine, Liverpool, L3 5QA UK; 5 School of Public Health, Faculty of Health Science, University of the Witwatersrand, Johannesburg, South Africa; 6 School of Life Science and Biotechnology, Nelson Mandela African Institution of Science and Technology, Arusha, Tanzania; 7 Wits Research Institute for Malaria, Faculty of Health Sciences, University of the Witwatersrand, Johannesburg, South Africa; 8 Centre for Emerging Zoonotic Parasitic Diseases, Vector Control Reference Laboratory, National Institute for Communicable Diseases of the National Health Laboratory Service, Johannesburg, South Africa

**Keywords:** *Anopheles funestus* group, Species identification, Insecticide resistance, Population genetics, Gene flow, Genetic control, Malaria, Vector surveillance

## Abstract

*Anopheles* mosquitoes present a major public health challenge in sub-Saharan Africa; notably, as vectors of malaria that kill over half a million people annually. In parts of the east and southern Africa region, one species in the Funestu*s* group, *Anopheles funestus,* has established itself as an exceptionally dominant vector in some areas, it is responsible for more than 90% of all malaria transmission events. However, compared to other malaria vectors, the species is far less studied, partly due to difficulties in laboratory colonization and the unresolved aspects of its taxonomy and systematics. Control of *An. funestus* is also increasingly difficult because it has developed widespread resistance to public health insecticides. Fortunately, recent advances in molecular techniques are enabling greater insights into species identity, gene flow patterns, population structure, and the spread of resistance in mosquitoes. These advances and their potential applications are reviewed with a focus on four research themes relevant to the biology and control of *An. funestus* in Africa, namely: (i) the taxonomic characterization of different vector species within the Funestu*s* group and their role in malaria transmission; (ii) insecticide resistance profile; (iii) population genetic diversity and gene flow, and (iv) applications of genetic technologies for surveillance and control. The research gaps and opportunities identified in this review will provide a basis for improving the surveillance and control of *An. funestus* and malaria transmission in Africa.

## Background

Malaria transmission is driven by female *Anopheles* mosquitoes. In Africa, the four major malaria vectors are *Anopheles gambiae, Anopheles coluzzii, Anopheles arabiensis,* and *Anopheles funestus *sensu stricto (*s.s*.). Different *Anopheles* species exhibit contrasting behaviours and physiological responses to interventions, such as insecticide-treated nets (ITNs) and indoor residual spraying (IRS), which are the core World Health Organization (WHO)-recommended vector control methods [1]. For instance, *An. gambiae, An. coluzzii,* and *An. funestus s.s.* predominantly prefer blood-feeding on humans (anthropophilic) and resting indoors (endophilic) [2, 3]*,* whereas *An. arabiensis* is a more generalist vector, biting humans but also readily feeding on domestic animals (zoophilic) and resting both outdoors (exophilic) and indoors [4]. However, some evidence indicates that *An. funestus s.s.* is shifting resting and feeding behaviour to the outdoors [5] and sometimes during daytime in some settings as an adaptation to indoor interventions [6]. Models predict such behavioural shifts coupled with vector resistance could have devastating public health outcomes [7]. Additionally, such innate or evolving differences in behaviours between vector species mean that interventions can have differential effects depending on local vector ecology.

*Anopheles funestus s.s*., hereafter referred to as *An. funestus*, is emerging as one of the most important malaria vectors and is increasingly dominant even in areas where it co-occurs with the other prominent vector species*.* For instance, in certain parts of Tanzania, *An. funestus* contributes consistently more than 90% of the yearly entomological inoculation rate (EIR) even in areas where it is far less abundant than *An. arabiensis* [8–11]*.* A similar trend of vectorial dominance has been observed in Cameroon where the vector contributes over 70% EIR in certain villages [12, 13], in Burkina Faso where it seasonally dominates transmission [14], and in Malawi [15], Zambia [16], and Kenya [17] where it is established as the primary malaria vector. Given the observed competence of *An. funestus* in the East and Southern Africa and in parts of Central and West Africa, it might be reasonable to generate a continent-wide comparative assessment on the importance of *An. funestus* in malaria transmission in different settings.

**Fig. 1:**
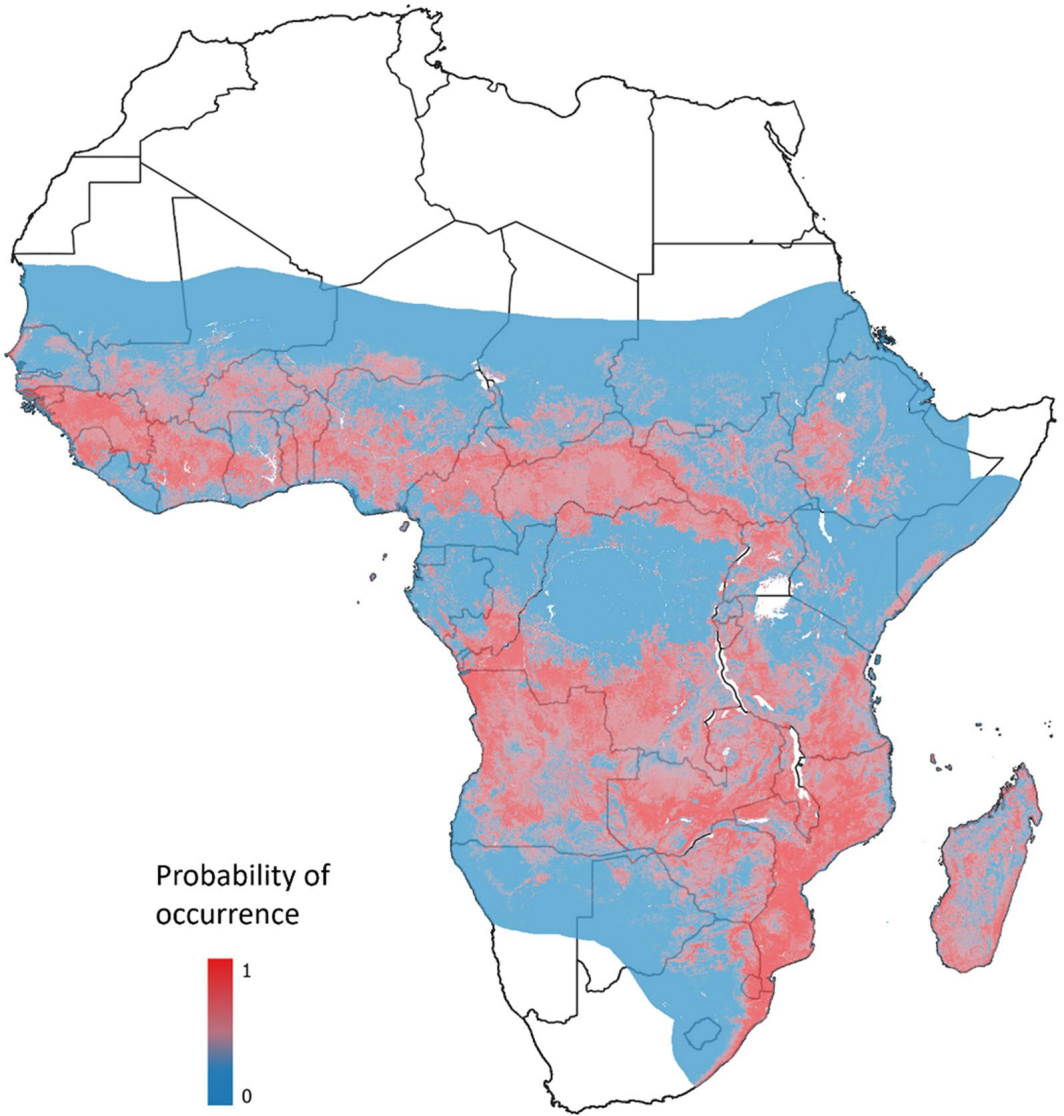
The distribution of the *Anopheles funestus* group in Africa. The red areas indicate countries with the confirmed presence of at least one member in the group while the blue area indicates areas where the species is not yet recorded. The map was created in R software (v 4.2.1) and QGIS (v 3.18) using raster data sourced from the Malaria Atlas Project (https://malariaatlas.org/)

*Anopheles funestus* belongs to the *An. funestus* group of mosquitoes, hereafter referred to as AFG, which has at least 10 other African sibling species, namely: *An. funestus-like, Anopheles parensis, Anopheles vaneedeni, Anopheles aruni, Anopheles confusus, Anopheles leesoni, Anopheles longipalpis, Anopheles rivulorum, An. rivulorum-like, Anopheles brucei,* and *Anopheles fuscivenosus* [18]. Some of these species are morphologically indistinguishable at the adult stage, though experienced entomologists can distinguish some species at the aquatic stage using identification keys [18]. Collectively, the AFG mosquitoes have a wide geographical distribution across sub-Saharan Africa (Fig. 1). *Anopheles funestus, An. leesoni* and *An. rivulorum* occur in most tropical Africa, while *An. parensis, An. confusus,* and *An. aruni* are localized to eastern African countries. *Anopheles vaneedeni, An. parensis*, *An. fuscivenosus, An. funestus*-like and *An. longipalpis* are native to southern African countries, while *An. rivulorum-like* and *An. brucei* are also frequent in West and Central Africa [19–21]. Within the AFG, *An. funestus* is considered the main malaria vector across Africa due to its preferentially anthropophilic nature [22] and having the highest malaria infection rates [10]. However, other members in this group have also been shown to be naturally infected by malaria parasites. For instance, *An. rivulorum* were observed to be active early in the night, outdoors, and were found to be carrying *Plasmodium falciparum* in Tanzania and Kenya [23, 24]. Similar observations have been made on *An. longipalpis* in Kenya [25], *An. vaneedeni* in South Africa [26], and *An. parensis* in Uganda [27] and South Africa [28] where they are considered to contribute to residual malaria transmission. While *An. funestus* is likely the most important vector in this group in most settings, others may play a role as secondary vectors. Thus, it is important to ensure accurate and robust methodologies to distinguish members of this group as required to assess their relative contribution to residual transmission.

In many countries, malaria vector control is also increasingly difficult because of widespread resistance to public health insecticides [29]. Compared to other malaria vectors, *An. funestus* populations are increasingly surviving diagnostic doses of pyrethroids in bioassays [30]. Experimental work has shown that the increasing resistance in this vector significantly reduces the effectiveness of ITNs [31]. Despite the elusiveness of quantifying the impact of vector resistance on malaria epidemiology at the community level [32], recent data from an area with highly resistant *An. funestus* as the primary vector [8, 33] indicates the superiority of dual active ITNs compared to standard ITNs in reducing malaria cases, suggesting a key role of resistant *An. funestus* in malaria transmission [34]. As physiological resistance continues to spread in Africa, it is important to understand its underlying mechanisms to inform more effective control strategies. Since the spread of resistance genes in mosquito populations is influenced by gene flow [35], population genetics using whole genome sequencing provides unique insights into the structure of malaria vectors, as recently shown in *An. gambiae* [36]. This approach also opens avenues for discovering new resistance mechanisms [37] and could generate essential information for the development and deployment of genetic control tools such as gene drives [38].

Previous reviews have outlined recent advances in characterizing chromosomal inversion and the development of PCR molecular diagnostic assays for *An. funestus* [39], and the advances in insecticide resistance profiling and population genetics of *An. funestus* [40]. However, despite a significant increase in research on these topics over the last ten years, there has not been a recent review publication of current knowledge on the molecular basis of insecticide resistance, gene flow patterns, and fine-scale population structuring of *An. funestus*. This is warranted as advances in molecular techniques in the last decade can enable greater insights into the real identity of vector species and how the resistance genotypes are spreading within and between mosquito population clusters. There have also been recent improvements in the *An. funestus* reference genome [41, 42], which can provide newer insights into the genetic basis of the mosquitoes' behavioural and physiological attributes.

A selection of these advances and their potential applications are reviewed along four key research themes relevant to the biology and control of *An. funestus* in Africa, as follows: (i) taxonomic and systematic characterization of the vector, its sibling species, and their roles in malaria transmission in different settings; (ii) improvements in insecticide resistance profiling and associated technologies; (iii) improved understanding of the genetic structure and gene flow patterns between and within population clusters of the vector species, and (iv) potential applications of genetic technologies for surveillance and control of the species. The review provides a basis for identifying key research gaps and opportunities for an R&D agenda relevant to the surveillance and control of *An. funestus* and malaria transmission.

## Molecular approaches to identify members of the *An. funestus* group

Morphological keys for the identification of members of the AFG and other African *Anopheles* mosquitoes were established between 1968 [43], then updated in 1987 [44], and more recently updated in 2020 with an improved description of many *Anopheles* species complexes and groups [18]. This method of identification requires well-trained taxonomists, and either field collection of aquatic stages that are reared to the adult stage, which takes between 2 and 4 weeks, or trap collection of adults. Even when samples suitable for morphological identification are available, some members of the AFG are challenging to distinguish taxonomically at either stage, necessitating the adoption of molecular methods (Table 1).

**Table 1:** Molecular diagnostic approaches to distinguish members of the *An. funestus* group

Diagnostic method	Approach	Members distinguished	Advantages	Limitations	References
PCR-RFLP-1	PCR primers developed from *An. funestus* D3 region of 28S ribosomal gene amplified and digested using restriction endonuclease *HpaII*	*An. funestus* and *An. vaneedeni*	Ability to distinguish two morphologically similar species	Limited to only two members of the *An. funestus* group Minimal sequence variation between the two species poses the challenge of specificity when other members are added to the assay Post PCR processing	[48]
Single-strand conformation polymorphism (SSCP) PCR	PCR primers developed from *An. funestus* D3 region of 28S ribosomal gene amplified and separated based on DNA conformation on SSCP gels	*An. funestus*, *An. vaneedeni*, *An. rivulorum*, and *An. leesoni*	Ability to distinguish four members of the An. funestus group Can distinguish east from West African *An. funestus*	Overlap on the denatured single strand (DSS) banding patterns for *An. funestus* and *An. vaneedeni* Post PCR processing	[49]
Internal transcribed spacer 2 (ITS2)	PCR amplification using internal transcribed spacer 2 (ITS2) region primers	*An. funestus* and *An. rivulorum*	Differentiating two sympatric vectors in the *An. funestus* group	Limited to only two members within the group Post PCR processing	[50]
ITS2 Cocktail PCR		*An. funestus, An. rivulorum, An. vaneedeni, An. leesoni, An. parensis, and An. rivulorum-like*	Simultaneous identification of six members within the group in a single PCR run	Post PCR processing	[51, 52]
PCR–RFLP-2	PCR amplification using AFG multiplex PCR assay followed by *Eco*RI RFLP digestion	*An. funestus, An. funestus-*like*, An. parensis, Anopheles rivulorum, An. vaneedeni, An. leesoni, and An. longipalpis-*type C	Identification of *An. longipalpis-*type C from the other members of the AFG	Post PCR processing and requirement of enzyme digestion	[128]

Before 1990, *Anopheles* identification was primarily done by a combination of morphological characterization [44] and cytogenetic analysis of chromosomal inversions [45–47]. Cytogenic analysis is however a tedious process, stage and sex-specific, and requires semi-gravid females [47]. Efforts to develop molecular diagnostics for the *An. funestus* group began in the 1990s with a polymerase chain reaction (PCR) coupled with restriction fragment length polymorphism (PCR–RFLP) targeting the 28S ribosomal gene to distinguish between *An. funestus* and *An. vaneedeni* [48]. This was later expanded to a single-strand conformation polymorphism (SSCP) assay, targeting the same gene, to distinguish *An. funestus*, *An. vaneedeni*, *An. rivulorum*, and *An. leesoni* [49]. Overlap in DNA banding patterns between the renatured and denatured single strands necessitated the development of a further assay, targeting ribosomal internal transcribed spacer 2 (ITS2), to distinguish the two medically important vectors, *An. funestus* and *An. rivulorum* [50]. This eventually laid the ground for the development of the AFG multiplex PCR assay, which is a cocktail PCR assay that can distinguish five members (*An. funestus, An. rivulorum, An. vaneedeni, An. leesoni,* and *An. parensis*) of the AFG in a single run [51]. However, an initial challenge observed with the AFG multiplex PCR assay, was that its performance was not generalizable to some parts of Africa, especially Central and West Africa where it could not readily identify *An. rivulorum* [52]. Further analysis revealed the presence of *An. rivulorum*-like as a separate species [52], which was later also confirmed in South Africa [21]. Similarly in Malawi, the AFG multiplex PCR assay failed to identify samples morphologically characterized into the AFG but were later designated as *An. funestus*-like by using cytogenesis and DNA-based methods [53]. Following these discoveries, the AFG multiplex PCR assay has since been expanded to include species-specific primers for *An. funestus*-like and *An. rivolurum*-like.

Given several challenges in AFG species identification, it is key to combine different approaches, such as morphological and molecular data that could be used together with geographical location data to infer the species. For example, although *An. leesoni* is closely related to the Asian vector *Anopheles minimus*, it is considered a separate species based on its geographical separation. Similarly, and indeed an often-neglected challenge is the inclusion of non-AFG samples in a diagnostic PCR assay, which may erroneously identify them as a member of the AFG [54]. For example, a member of the *Anopheles marshalli* group or *An. gambiae* complex mistakenly included in the AFG multiplex PCR assay would be erroneously identified as *An. leesoni* [54]). Therefore, careful sample handling and morphological identification are key to improving this analysis. Accurate species identification is also critical for epidemiological studies, for example, the malaria parasite was recently reported from *An. longipalpis* C, considered not to be a malaria vector [55], and a molecularly unidentified cryptic species within the AFG [23]; similarly to findings in cryptic species within the *An. gambiae* complex [56]. These new findings highlight the need to acknowledge mosquito species whose identity is not fully resolved as potential vectors and the need for field estimates of malaria transmission to incorporate the *Anopheles* species found in an area more broadly. Lastly, the employment of vector genomic surveillance, even on a small scale, should be established as part of routine national vector monitoring programmes. Country-level investments are, therefore, necessary to enhance training for control programmes to better integrate taxonomy and molecular techniques to improve species identification and incrimination.

## Population genetics of *An. funestus*

Understanding mosquito populations enables the assessment of the feasibility and potential impact of genetic control approaches such as gene drives for disease control. It also provides a basis for monitoring the spread of genetic traits such as insecticide-resistance alleles. Molecular techniques ranging from chromosomal inversions, mitochondrial DNA analysis, restriction fragment length polymorphisms (RFLP), microsatellite genotyping, and whole-genome sequencing (WGS) have been employed to provide insights into patterns of *An. funestus* population interactions and structuring [57–60]. The utility of these techniques in advancing studies in *An. funestus* population genetics are highlighted below.

### Chromosomal inversions

Chromosomal inversions in mosquitoes are important drivers of local adaptation to varying environmental factors [61, 62]. This is important for *An. funestus* that has both a wide geographical range across Africa (Fig. 1) and high levels of chromosomal polymorphisms [39]. The technique for studying inversions was developed in the 1970s and follows a process where the ovaries of half-gravid females are squashed, stained, and observed under a phase contrast microscope to reveal the polytene chromosomes [47], which are then scored using a chromosome map developed by Sharakhov et al. [63].

Costantini et al. first identified two chromosomal forms of *An. funestus* in Burkina Faso, namely Kiribina and Folonzo, based on 3Ra, 3Rb, and 2Ra inversions [60, 64]. The Kiribina form is distinguished by an inversion in the 2R and 3R chromosomes, while Folonzo has inversions 3Ra and 3Rb [60]. Both ecotypes are sympatric, highly anthropophilic, and contribute significantly to malaria transmission in their localities [64]. In Cameroon, Folonzo was found to occupy the equatorial zones of the country while Kiribina occupied the dry savannah regions [65]. Similarly, Ayala et al*.* found differentiation in chromosomal inversions in *An. funestus* collected from different ecologies in the same country [66] with the inversions causing a reduction in nucleotide diversity resulting in overall low genetic differentiation among the ecotypes [67]. In Angola, five inversions were detected, 2Ra, 2Rh, 3Ra, 3Rb, and 3La, with only samples from central Angola (Huambo province) designated as Folonzo chromosomal forms [68]. Taken together, these studies suggest an important association between chromosomal inversions and adaptation to diverse ecological conditions, which might be contributing to the spatial and temporal extension of malaria transmission [69].

Cytogenic karyotyping is still largely based on a tedious technique that requires highly skilled personnel and cannot be used on mosquito life stages other than gravid females, thus constraining the scalability of its use. Hence, a genotyping assay has recently been developed that utilizes tag single nucleotide polymorphisms (tag SNP) on the 3Ra, 3Rb, and 2Ra inversions to distinguish different *An. funestus* ecotypes [70]. This new method has a high agreement with the traditional cytogenic karyotyping method and can be deployed on any mosquito life stage, making it more convenient for high throughput cytogenic studies. However, this method has some limitations as current tag-SNPs are suitable only for distinguishing between the two West-African ecotypes of *An. funestus*, Folonzo and Kiribina. Additional innovation will be necessary to develop high-throughput techniques that can be used for broader analyses of chromosomal inversions across the continent; especially in an era where genomic sequence data are increasingly available. This will enable investigation of how these inversions might influence vector dispersal and adaptability in the face of climate change.

### Mitochondrial DNA analysis

**Fig. 2:**
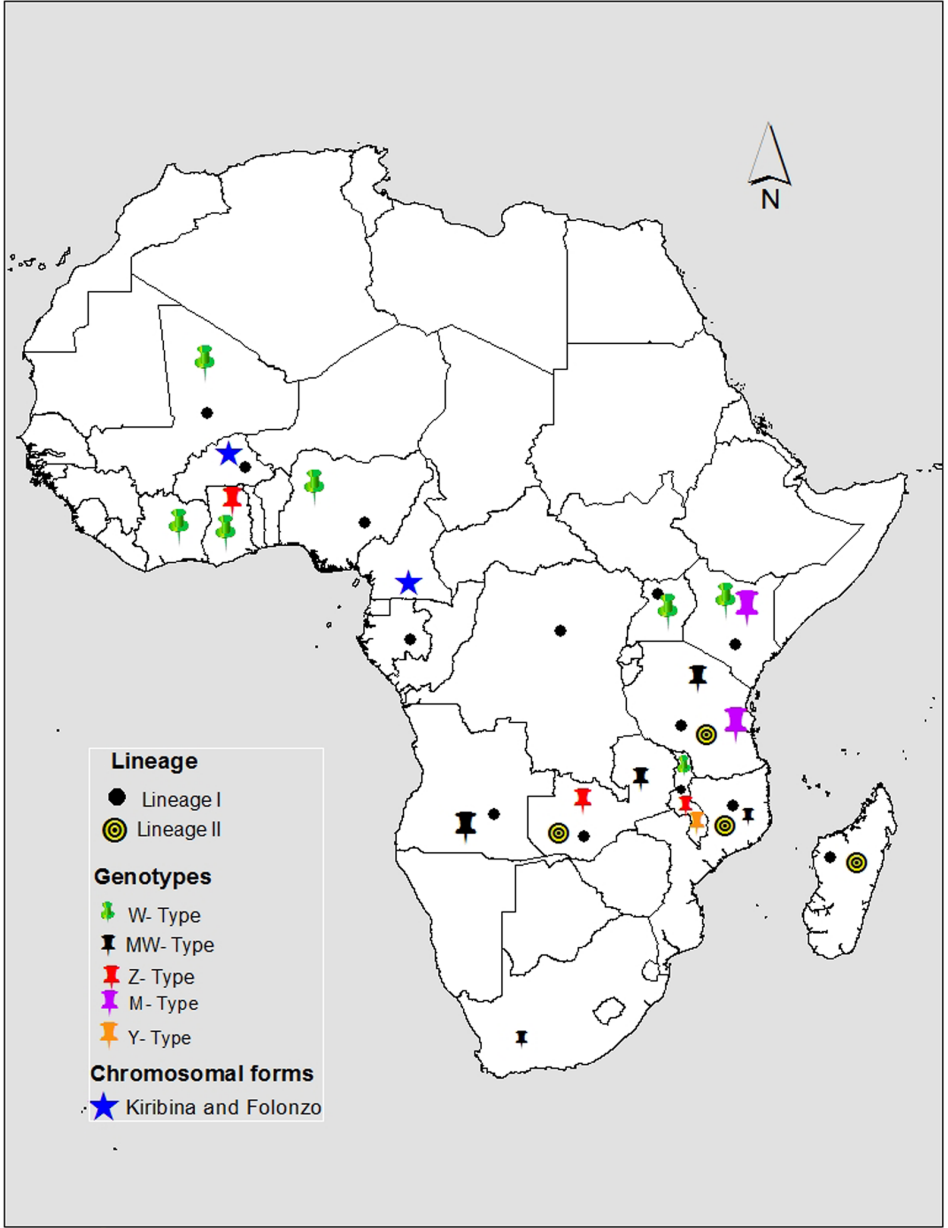
The distribution of *An. funestus*. **a** Lineages I and II [59, 73] **b** RFLP genotypes, W-type, MW type, Z-type, M-type, and Y-type [58] and **c** Chromosomal forms Kiribina and Folonzo [60]. The map was created using QGIS (v 3.18)

Analysis of mitochondrial DNA (mtDNA) is useful in reconstructing mosquito phylogenetic relationships, molecular evolution, and understanding their population history, i.e. time of taxon divergence [71]. For example, Michel et al. analyzed partial mitochondrial genes, NADH-ubiquinone oxidoreductase chain 5 protein (ND5) and Cytochrome c oxidase I (COI), from 11 countries across Africa, showing that *An. funestus* is genetically grouped into Eastern, Western, and Central populations, and detected two lineages I and II [59], defined as a group of mosquitoes that are ancestrally connected by using maternally inherited mitochondrial genes. Whilst lineage I was widespread across sub-Saharan Africa, lineage II was found restricted to mosquitoes from Tanzania, Madagascar, Zambia, and Mozambique [58, 59, 72] (Fig. 2). Similarly, Jones et al*.* [73] analysed 43 complete mitochondrial genomes of *An. funestus* from Zambia, the Democratic Republic of the Congo, and Tanzania, identifying 41 unique haplotypes, comprising 567 polymorphisms. This study also detected two distinct yet partially sympatric, lineages of *An. funestus*, lineage I and II, estimating their divergence to half a million years ago. An analysis of these lineages in the context of plausible introgression within the AFG indicates a genetic exchange between *An. parensis* and *An. funestus* before its rapid geographic range expansion [74]. Recently, a PCR-based diagnostic using hydrolysis probe analysis has been developed to identify these lineages in field-collected samples [75] that could be expanded to improve the identification of lineages in *An. funestus*.

### Restriction fragment length polymorphisms (RFLP) of ribosomal DNA

Geographical barriers to gene flow such as the Great Rift Valley have been hypothesized to influence how mosquitoes interact in space and time, shaping their population structure and adaptation. An RFLP analysis of a variable domain (D3) of the 28S ribosomal nuclear DNA in *An. funestus* mosquitoes sampled from either the eastern or western sides of the Rift Valley found different RFLP profiles; specifically W, M, and MW-types in the West, East, and Southern African countries respectively [58]. Notably, samples from Malawi, which is at the southern tip of the Rift Valley, had all the Y, Z, M, W, and MW genotypes (Fig. 2), suggesting that major landscape features could be guiding the directional flow and geographical convergence of genes in mosquito populations and shaping their genetic profiles. Natural barriers to gene flow other than the Rift Valley, such as wildlife reserves and forests, urban landscapes, lakes, and, mountain ranges, should also be investigated to assess their effect on *An. funestus* diversity.

### Microsatellite genotyping analysis

Once *An. funestus* microsatellite markers were physically mapped [63, 76], they became the indicator of choice for studying population diversity, gene flow patterns, migration rates, population size, bottlenecks, and kinship. These markers are robust due to their codominant nature, neutrality, random repeats across genomes, and conformity to Mendelian inheritance [77].

Continentally, microsatellite analysis shows that *An. funestus* subdivides into eastern, western, and central African genetic populations, broadly consistent with mitochondrial DNA patterns, but offering clearer resolution [59]. Genetic diversity studies in southern African countries have revealed finer scale variation. Barnes et al*.* analysed samples from Malawi, Zambia, and Mozambique and found strong North–South segregation within mosquitoes from Malawi and Zambia, indicating high levels of gene flow [78]. However, within Malawi, they also found high *F*_*ST*_ values between southern populations when compared to those in the north, indicating the presence of a gene flow barrier [78]. Analysis of additional *An. funestus* samples from Uganda and Zimbabwe later corroborated the same diversity observed in the southern African region [79]. In Kenya, *An. funestus* collected from the western part of the country were found to be genetically distinct from coastal populations [80]; similar to the pattern observed previously using chromosomal inversion analysis [81]. These studies implicate the Rift Valley as a possible barrier to gene flow between populations. Genetic structuring has also been observed within and between island *An. funestus* populations in Comoros and Madagascar [82]. Ayala et al*.* found samples from the two islands to cluster separately but also observed in-country structure within Madagascar, which might be driven by landscape features. As *An. funestus* undergoes speciation across the continent, the role of these observed diversities should be investigated on how they influence vector adaptation, dispersal, and potential vectorial capacity.

### Whole genome sequence analysis

Currently, WGS studies investigating population diversity in *An. funestus* are rare in contrast with the extensive investigation that has been done on the *An. gambiae* complex [36, 56]. However, this is likely to change rapidly given the rise of collaborative programs such as the recent inception of the MalariaGEN *Anopheles funestus* genomic surveillance project (https://www.malariagen.net/projects/anopheles-funestus-genomic-surveillance-project) which is sequencing samples of this species from across Africa. A recent success of this program is the improved genome assembly from an individual female *An. funestus* (specimen from Gabon; 251 megabases) complete with the mitochondrial genome (15.4 kilobases) [41].

Genomic analysis has cross cutting potential of providing new insights into the genetic diversity of mosquito vectors, improving the surveillance of vectors and insecticide resistance, and providing an open resource for the development of new control tools. For example sequence analysis of *An. funestus* collected across Africa has shown Southern African mosquitoes to cluster separately from other African populations [57] with a similar clustering pattern found using microsatellite analysis suggesting a barrier to gene flow [83]. Several WGS in the *An. gambiae* complex have shown its potential. For example, genome-wide sequence analysis in the *An. gambiae* complex showed that *An. coluzzii* is largely restricted to West Africa whilst West, Central, and Eastern *An. gambiae* are genetically similar [56]. Additionally, WGS in the *An. gambiae* complex has revealed new mechanisms of insecticide resistance and associated alleles such as copy number variants [37].

Thus, WGS analysis on *An. funestus* has the potential of discovering fine-scale population structuring which is vital for elucidating population history and the spread of insecticide-resistance genes across the region. Unlike other approaches such as the use of microsatellites, WGS can allow delineation of more recent genetic changes in a population. However, from a practical perspective, simpler options involving specific amplicons are likely to be more widely used given their quicker turnaround times. WGS will remain an important research tool, but is unlikely to be practiced at scale.

## Insecticide resistance profiling in* Anopheles funestus*

Five classes of insecticides are recommended for the control of adult *Anopheles* vectors: pyrethroids, carbamates, organophosphates, organochlorines, and neonicotinoids. Given the limited options within these classes, and the widespread insecticide resistance [29], control programs need integrated measures to monitor phenotypic insecticide susceptibility and underlying resistance mechanisms to select appropriate insecticide products for the management of resistance.

Bioassays to determine phenotypic resistance are conducted following either the WHO or Centres for Disease Control and Prevention (CDC) bottle assays; in which mosquitoes are exposed to standard concentrations of an insecticide and either mortality or knock-down measured after a specific time [84]. The mechanisms of resistance responsible for resistance phenotypes include metabolic detoxification [85], target site mutations [86], cuticular thickening which reduces insecticide penetration [87], or behavioural avoidance to reduce exposure to insecticides [88].

### Metabolic resistance

Metabolic resistance occurs when a mosquito produces high levels of detoxifying enzymes that chemically modify and deactivate the insecticides. Three families of metabolizing enzymes are associated with resistance: esterase, monooxygenases, and glutathione-S-transferases (GSTs) [89].

Increased expression of monooxygenase enzymes belonging to the cytochrome P450 gene family (*CYP450*) is the most common cause of resistance to pyrethroids in *An. funestus* [31, 57, 85, 90, 91] Table 2. The expression of these genes varies considerably across Africa reflecting possible barriers to gene flow amongst populations [31, 57, 78]. For instance, the *CYP450* genes *CYP6P9a, CYP6M7,* and *CYP6P9b* are overexpressed (i.e., exhibit elevated transcription compared to susceptible strains) in *An. funestus* resistant populations in southern African populations from Zambia, Malawi, and Mozambique [31, 91, 92]. In contrast, the *CYP450* genes *CYP6M4, CYP9K1, CYP6P9b, CYP304b1, CYP6N1, CYP6M1, CYP6Z1,* and *CYP6M7* are overexpressed in resistant populations from Uganda and Tanzania in East Africa [93–95]. Recently, *CYP6Z1* has also been shown to confer carbamate and pyrethroid cross-resistance in the laboratory [96] though further field evaluation is needed.

**Table 2:** Selected genes involved in insecticide resistance in *An. funestus* and their geographical distribution across Africa

Geographical distribution	Insecticide class	Resistance mechanism	Resistance genes	References
Southern Africa	Pyrethroid	Metabolic	*CYP6P9a, CYP6M7,* and* CYP6P9b*	[31, 91]
Eastern Africa	Pyrethroid	Metabolic	*CYP6M4, CYP9K1, CYP6P9b, CYP304b1, CYP6M7, CYP6N1, CYP6M1,* and *CYP6Z1*	[93–95]
West Africa	Pyrethroid and Organochloride	Metabolic	*CYP6P9a, CYP6P9b,* and* GSTe2*	[97–99]
Central, South and West Africa	Organochloride	Metabolic and target-site	*CYP6P9a/b, A296S RDL,* and* GSTe2*	[99, 100]

In West Africa (Benin and Nigeria), the *CYP450 CYP6P9a, CYP6P9b,* and a glutathione S-transferase gene family (*GSTe2*) are overexpressed in resistant *An. funestus* mosquitoes and confer pyrethroid and dichlorodiphenyltrichloroethane (DDT) cross-resistance [97–99]. However, overexpression of *CYP6P9a* was not subsequently detected in West African samples in an analysis by Weedall et al*.* [58], indicating this mechanism may be restricted to East and Southern African countries. Similarly, overexpression of *CYP6P4a* appears to be restricted to Ghana, and *CYP6P5* overexpression is only found in East and West African samples [57]. In East and West African *An. funestus* populations overexpression of *CYP6P9a/b,* and glutathione S-transferase epsilon *(GSTE-L119F)* genes confer resistance to DDT [99, 100]. Contrastingly, in southern African countries, *An. funestus* populations remain susceptible to DDT insecticides [101, 102]. This demonstrates how diverse the genes involved in metabolic resistance across the continent are with *CYP6P9a/b* having a continental distribution.

Despite the crucial role of *CYP450* and *GSTs* genes in mediating insecticide resistance in *An. funestus*, simplified and field-applicable DNA-based assays for tracking metabolic resistance have not been available for this species. An assay for field tracking *CYP6P9a* in *An. funestus* has only recently been developed [31]. The assay is based on PCR–RFLP where a *Taq* I restriction enzyme cuts a 450 bp region of *CYP6P9a-*resistant mosquitoes but not in susceptible ones, allowing the distinction [31]. Such assays can easily advance monitoring of resistance alleles in the field without requiring sophisticated equipment, however, they might not be straightforward to design, depending on the genetic basis of the resistance. Considering the ubiquity of these resistance genes, the DNA-based assays should have a multigene panel approach where the most common genes can be amplified in the same assay.

### Target site resistance

Target-site resistance is caused by point mutations in insecticide-binding proteins which thereby inhibit the binding and toxic activity of the insecticide. The most widely studied target site mutation is knockdown resistance (*kdr*), which is based on a point mutation changing leucine to phenylalanine or serine at codon 1014 (995 using *An. gambiae* codon numbering) of the voltage-gated sodium channel (*VGSC*) in *An. gambiae* mosquitoes. The mutation reduces sensitivity to pyrethroids and DDT [103]. However, analysis of *VGSC* gene at the *1014* codon has not detected any mutation in *An. funestus* [104, 105]. This suggests that *kdr* might not be involved in DDT and pyrethroid cross-resistance in *An. funestus* [86, 103]. Other non-synonymous mutations in the *VGSC* gene in *An. funestus* such as I877L, V881L, and A1007S have been detected, and though require further investigation, do not appear to have a substantial impact on insecticide resistance [105].

Despite dieldrin not currently being used for vector control, *An. funestus* resistance to this insecticide remains high, especially in Central and West Africa. This is caused by an A296S-*rdl* mutation in γ-aminobutyric acid (GABA)-gated chloride channel in *An. funestus* from West Africa (Burkina Faso), Central Africa (Cameroon), and Southern Africa (Malawi), but not in East Africa [15, 106, 107] (Table 2). The mutation is likely to persist in the population even in absence of selection pressure due to its chromosomal location, that is close to the centromere, which reduces any cross-over event [107, 108]. Organophosphate resistance in *An. gambiae* and *An. coluzzii,* is driven by a *G280S/G119S* *ace*-1 mutation [109, 110], with little known in *An. funestus.* There is a need for research to understand the resistance mechanism of the commonly used IRS insecticide (pirimiphos-methyl), even though it remains efficacious against *An. funestus* [111].

The evolution of resistance in *An. funestus* populations primarily through metabolic resistance mechanism via P450s, makes them liable to cross-resistance with other insecticides. Looking into the future, the use of the WGS approach has the potential of discovering novel resistance mechanisms in *An. funestus* whilst also providing new insights into genes already implicated in resistance. In *An. gambiae* and *Aedes aegypti*, for instance, this technique has led to the discovery of a new resistance mechanism through gene duplication or copy number variation (CNV) in metabolic resistance genes [37, 112]. Copy number variations can lead to resistance, as the presence of more copies of a detoxifying gene will result in its overexpression [113]. As WGS costs are decreasing, its application for *An. funestus* resistance monitoring should be prioritized.

## The potential of genetic technologies for the surveillance and control of *An. funestus*

Genetic manipulation of disease vectors involves the deliberate release of individuals containing a desirable genetic trait to spread it through the wild-type population via mating [114]. Such approaches can include either population suppression through the spread of genes reducing vector reproduction, or modification of vector by introducing genes that confer refractoriness to pathogens [115]. Many of these gene drive approaches are based on the use of CRISPR-Cas9-based elements that can copy themselves from one chromosome to another in the germline and thereby increase their representation among the gametes. This type of approach ensures accuracy and super Mendelian inheritance leading to a rapid increase in the frequency of the desired traits in the target population [116–119].

The development of CRISPR-based genome editing tools and gene drives in *Anopheles* mosquitoes rapidly advanced over the past six years albeit with a focus on mosquitoes of the *An. gambiae* complex [38, 116]*.* However, a more holistic gene drive programme for malaria control will need to equally target the increasingly important African malaria vector, *An. funestus*. Fortunately, the transgenic pipelines and technologies that are already established for *An. gambiae* can be adapted to this species.

Only a handful of studies have been published that demonstrate the use of gene editing technologies in *An. funestus* [120, 121]. Using CRISPR/Cas9, Li et al*.* [124] showed for the first time that heritable germline mutations could be introduced into *An. funestus* genome by microinjection of guide RNAs (gRNAs) and Cas9 protein into eggs. This resulted in a stable colony that can be used for reverse genetics studies. This was achieved through a nonhomologous end-joining (NHEJ) repair process, following Cas9 cleavage of a target site that is determined by the sequence of the gRNA, which leads to small insertions or deletions. Quinn et al*.* [123] have also recently demonstrated the successful use of homology-directed repair (HDR), also known as knock-in, for the generation of transgenic *An. funestus*. HDR has the advantage of introducing the desired transgenic DNA sequence that is incorporated into the mosquito germline during the repair process. Since the copying mechanism of HDR is like what many of the CRISPR-based gene-drive rely on to increase their copy number, the high rates of HDR observed in *An. funestus* to date augur well for its amenability to gene drives of this type.

Assuming successful development of gene-drive constructs for *An. funestus*, several entomological and regulatory questions will need to be addressed before large-scale deployment. A major challenge is the mass rearing of modified *An. funestus* mosquitoes under laboratory settings. Currently, there are only two colony lines of the vector successfully established in the laboratory, *An. funestus* from Mozambique (FUMOZ) and *An. funestus* from southern Angola (FANG) [122, 123]. This is mainly due to the bottlenecks of adapting *An. funestus* into a laboratory colony which includes larval survival, mating success in cages, and low adult survival rates [124]. Overcoming these challenges will be key to establishing transgenic colony lines in the laboratory for experimentation and large-scale vector control use.

Additionally, the high levels of genetic diversity within *An. funestus* populations across Africa could impact the application of gene drive control strategies [57, 59, 78]. Sex-linked gene drive approaches are dependent on gene flow which is shaped by natural barriers such as large water bodies, large forests cover, aridity, valleys, and mountains [36]. Hence, fine-scale population genetics surveys of populations at target release sites must be undertaken as an integral part of the deployment strategy. Overall, the application of gene drives will need to be tailored depending on the local vector population and environmental and geographical features [125]. Similarly, it will be vital to resolve regulatory issues around ethics, and environmental impact, and importantly allow the communities living in malaria-endemic areas to have a leading voice in the development and deployment of such tools [126, 127].

## Conclusion

The last decade has seen an upsurge in *An. funestus* group research to understand mechanisms of insecticide resistance, taxonomy, and population biology. A combination of robust morphological identification, allele-specific PCR, and small-scale WGS should be used in tandem with geographical information when profiling mosquito identity. Cytochrome *P450-*mediated metabolic resistance starkly varies across the continent hence the development of field adaptable DNA-based assay to diagnose it should be a priority to help in resistance management and surveillance. Similarly, a detailed analysis of *An. funestus* population genetics should be undertaken on how it influences the spread of resistance genotypes and as a prerequisite to the deployment of genetic control tools. Attempts at malaria control and elimination need to be holistic bringing together current and emerging vector control approaches, a pool of empowered human personnel, and most importantly involving communities who bear the burden of this disease.

## Data Availability

Not applicable.

## Abbreviations

AFG: *An. funestus* Group
WHO: World Health Organization
IRS: Indoor residual spraying
ITN: Insecticide treated net
PCR: Polymerase chain reaction
RFLP: Restriction fragment length polymorphism
WGS: Whole-genome sequencing
NHEJ: Nonhomologous end-joining
HDR: Homology-directed repair
